# Effect of root exudates of *Eucalyptus urophylla* and *Acacia mearnsii* on soil microbes under simulated warming climate conditions

**DOI:** 10.1186/s12866-019-1604-6

**Published:** 2019-10-15

**Authors:** Jiahui Wu, Shixiao Yu

**Affiliations:** Department of Ecology, School of Life Sciences /State Key Laboratory of Biocontrol, Sun Yat-sen University, Guangzhou, 510275 China

**Keywords:** *Acacia mearnsii*, Climate change, *Eucalyptus urophylla*, Root exudates, Soil microorganisms

## Abstract

**Background:**

Recent studies demonstrated that warming and elevated carbon dioxide (CO_2_) indirectly affect the soil microbial community structure via plant root exudates. However, there is no direct evidence for how the root exudates affect soil microbes and how the compositions of root exudates respond to climate change.

**Results:**

The results showed that warming directly decreased biomass of soil-borne bacteria and fungi for *Acacia mearnsii* De Willd but it did not impact soil microbial community for *Eucalyptus urophylla S.T. Blake*. In contrast, elevated CO_2_ had strong direct effect on increasing soil microbial biomass for both plant species. However, plant roots could significantly increase the secretion of antibacterial chemicals (most probable organic acids), which inhibited the growth of bacteria and fungi in elevated CO_2_ environment. This inhibitory effect neutralized the facilitation from increasing CO_2_ concentration on microbial growth.

**Conclusions:**

We concluded that climate change can directly affect microorganisms, and indirectly affect the soil microbial community structure by changes in composition and content of plant root exudates.

## Background

The global climate is predicted to change drastically over the next century. According to IPCC (2013), the global surface temperature may increase by 1.8–4.0 °C at the end of the twenty-first century, driven by increased atmospheric CO_2_ levels derived from natural and/or anthropogenic sources [1]. The response of terrestrial ecosystems to global climate change has received much attention and several comprehensive reviews have shown that atmospheric CO_2_ enrichment will likely increase plant biomass and forest production, based on increase net carbon assimilation and water use efficiency of most plants [2–4]. Additionally, global warming is expected to affect soil processes in ecosystems, including soil respiration, nutrient cycling and the decomposition of SOM [5–7]. It has been widely recognized that soil microorganisms are the driving factors of biogeochemical processes, which play a key role in the response of soil ecosystems to the rising atmospheric CO_2_ and temperature because they mineralize organic matter and drive nutrient cycling [8–10]. Understanding how global warming will influence soil community composition and biodiversity will be needed before it will be possible to predict the functioning of natural and managed ecosystems [11, 12].

Plenty of experimental results have shown that global warming has direct impacts on soil microbial respiration, activities, composition and as well as the process rates [4, 5, 12, 13]. However, there is also evidence of indirect effects of climate warming, such as affecting plant photosynthesis and respiration, changing the ratio of carbon and nitrogen nutrition in plants and associated root exudates inputs to the rhizosphere, witch lead to changes in soil microbial community structure [14–16]. Therefore, understanding how rising atmospheric CO_2_ and temperature affects the microbial properties through root exudation could provide a new perspective for understanding the feedback of terrestrial ecosystems to climate warming.

Root exudates, a wide range of chemical compounds secreted by plant roots, have been known to act as the key mediator for the belowground interactions and functions of plant-microbe-soil system [17, 18]. Many studies have shown that the quantity and quality of root exudates determine the amount and type of rhizosphere microorganisms, and affect microorganism growth and metabolic processes [19]. Through the exudation of a wide variety of compounds, roots may regulate the soil microbial community both positively and negatively. The positive interactions include symbiotic associations with beneficial microbes, such as mycorrhiza and plant growth promoting rhizobacteria (PGPR). Negative interactions mostly include antimicrobial effect [20, 21]. There was enormous progress during the last two decades in the characterization of factors determining root exudation, which exhibits high variability among different plant species and even cultivars, within different root zones and developmental stages of individual plants and in response to various biotic and abiotic stress factors [17, 22]. Therefore, the changes in root exudate concentration, and their influences on soil microorganism are unpredictable under elevated CO_2_ and warming condition. *Eucalyptus* spp. and *Acacia* spp. have been introduced and widely planted in many countries throughout the world because of their high productivity, wide adaptability, and rapid economic returns. However, the proliferation of Eucalyptus plantations in south China has produced many problems for the local environment. The major one being the plant biodiversity reduction, which many studies have shown that the allelopathic effects of Eucalyptus are the main cause [23, 24]. As plant root exudates are a major source of allelopathic chemicals, a number of experiments have focused on the effects of root exudation on the early growth stages of various receptor species, like seed germination, seedling emergence and growth [15, 23], few studies focused on the effects of allelopathic chemicals of Eucalyptus on soil microbes [25]. Meanwhile, *Acacia* species is known to be highly invasive, threatening the ecology of a broad range of habitats by competing for nutrients and water, replacing grass communities and reducing native biodiversity [26, 27]. As known that the soil microbes is a key factor of the invasive character [28, 29], the understanding of the relationship between root exudates of *Acacia* species and native soil microbial structure is thus crucial to understand the process of invasion. In addition, this will contribute to develop ecological strategies aiming at reducing the negative impact of such invasive species and improving the restoration of native habitats.

Although root exudation is increasingly recognized as an important contributor to soil microbial process [18, 19], related studies about the impact of compounds variation of root exudation on soil microbial community structure in the wake of climate warming are still lacking, especially for some widely cultivated tree species. In this study, we selected two widely planted plantation trees, *Eucalyptus urophylla* S.T. Blake and *Acacia mearnsii* De Willd, as target species and designed a long-term pot experiment to explore the following: (1) how the microbial community structure and composition as well as plant root exudates respond to elevated temperature and CO_2_ concentration and (2) how the changes in root exudate compounds and concentration affect soil microorganisms.

## Results

### Identification and quantification of *A. mearnsii* root exudates

**Fig. 1:**
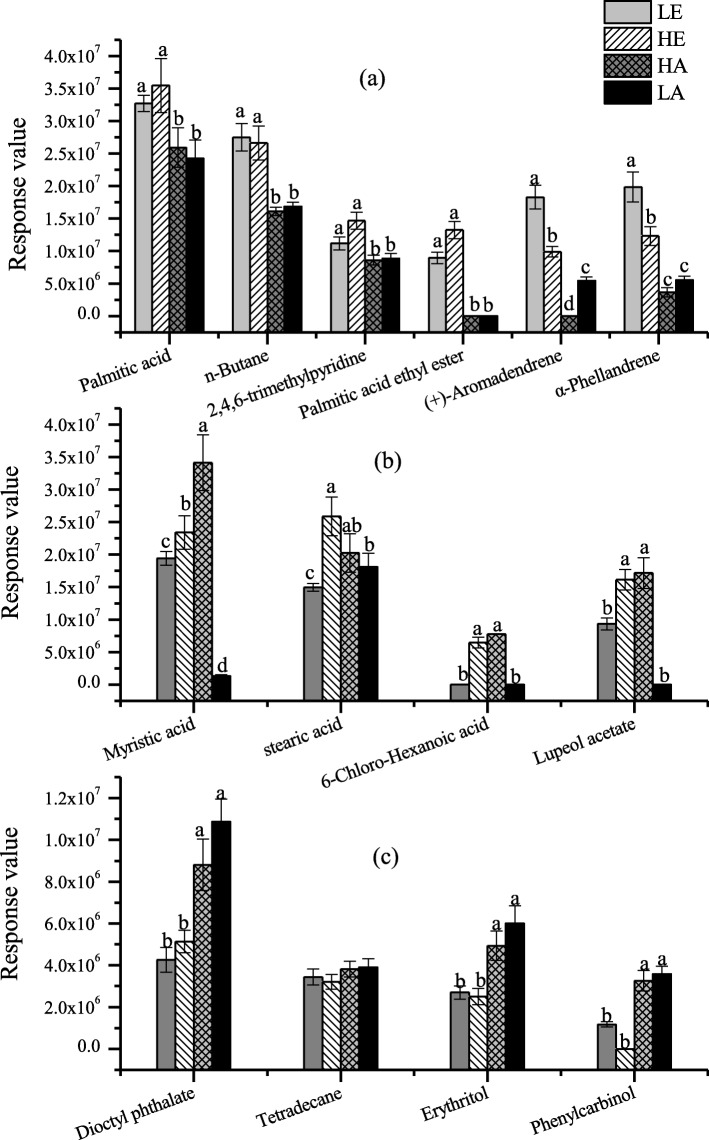
Three type compounds of *Acacia mearnsii* root exudates. **a **The first category: more secretion under elevated CO_2_ condition, **b** The second category: more secretion under higher temperature condition, and **c** The third category: more secretion under ambient CO_2_ condition. The mean values and standard deviations of three replicates are presented. Significant differences of variable means among different treatments at each sampling date are indicated by different letters (*P* < 0.05). Legends refer to Table 1

The GC-MS data resulted in 39 compounds being identified from root exudates of *A. mearnsii* under four environmental conditions (Additional file 1: Table S1); 19 compounds coexisted in four environmental conditions but their relative contents differed significantly. The PCA divided the root exudates into three categories (Additional file 1: Figure S1). The first category of compounds in *A. mearnsii* root exudates is shown in Fig. 1a. Significantly more of these compounds existed under elevated CO_2_ conditions – occurring for 18 compounds. Organic acids and terpenoids were dominant compounds, which occupied 45.65 and 20.2% of root exudates, respectively. Palmitic acid, n-butane, Palmitic acid ethyl ester, (+)-Aromadendrene, α-Phellandrene and 2,4,6-trimethylpyridine were relatively abundant. Experimental warming promote the secretion of four compounds (Fig. 1b), including stearic acid, 6-chloro-hexanoicacid, lupeol acetate and myristic acid. The relative abundances of these compounds were 4.63% (LA), 3.72% (LE), 18.26% (HA) and 19.62% (HA), respectively. The third category is shown in Fig. 1c. Relative contents of these compounds were significantly higher under lower CO_2_ conditions, including of Dioctyl phthalate, Tetradecane, Erythritol.

### Identification and quantification of *E. urophylla* root exudates

**Fig. 2:**
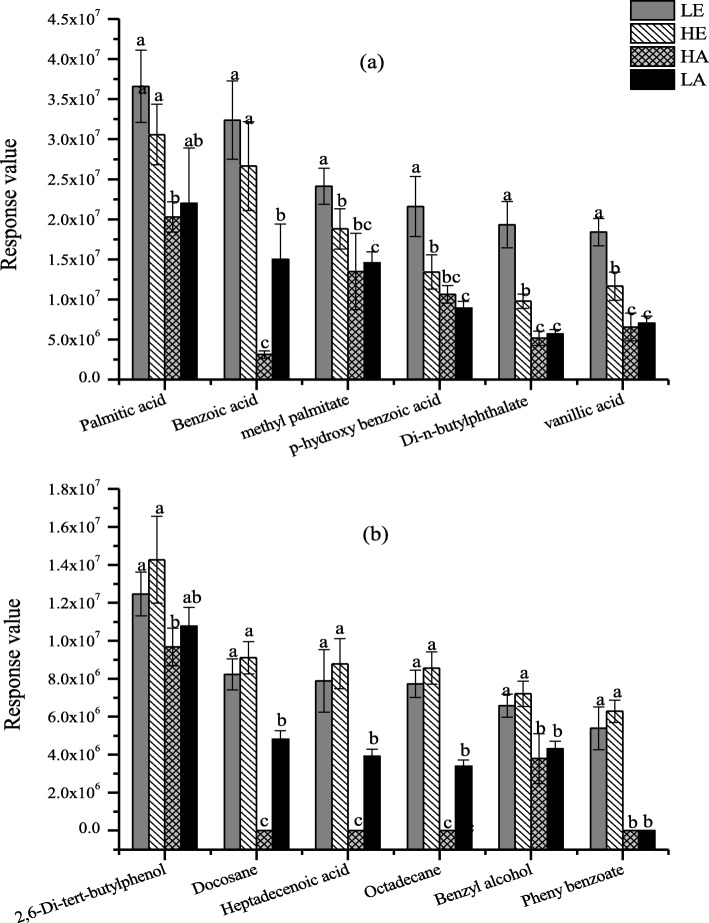
Two type compounds of *Eucalyptus urophylla* root exudates. **a** The first category: more secretion under elevated CO_2_ condition, **b** The second category: more secretion under higher temperature& elevated CO_2_ condition. The mean values and standard deviations of three replicates are presented. Significant differences of variable means among different treatments at each sampling date are indicated by different letters (*P* < 0.05)

The GC-MS data resulted in identification of 35 compounds from root exudates of *E. urophylla* under four environmental conditions (Additional file 1: Table S2). Of these, 21 compounds coexisted in four environmental conditions, but their relative abundance were significantly different. Two categories of root exudates were classified based on PCA analysis (Additional file 1: Figure S2). As shown in Fig. 2a. The relative contents of these 18 root secretions were significantly higher under elevated CO_2_ conditions. Organic acids, ester and alkane were dominant compounds, which including palmitic acid, benzoic acid, methyl palmitate, p-hydroxy benzoic acid, di-n-butyl phthalateare vanillic acid, methyl geranate and n-heneicosane. The second category of compounds in *E. urophylla* root exudates is shown in Fig. 2b. This category had a similar trend to the first category, but in this case the relative contents were highest in warming combined with elevated CO_2_ treatment, including 2,6-Di-tert-butylphenol, docosane, heptadecenoic acid, octadecane and benzyl alcohol.

### PLFA analysis of soil microorganisms

Our results showed that elevated CO_2_ had a significant direct impact (bare soil treatment) on total microbial PLFAs, Gram-positive bacteria PLFAs (G^+^), Gram-negative bacteria PLFAs (G^−^), fungal PLFAs (F) and actinomycetes PLFAs (A) at both *E. urophylla* (Additional file 1: Figure S3a) and *A. mearnsii* (Additional file 1: Figure S4a) soil. In LE condition, G^+^, G^−^ and F of *E. urophylla* soil were 5, 3, 2, 12 times higher than in LA condition, respectively. Similar result found in *A. mearnsii* soil, total PLFAs and PLFAs of various microorganisms were also higher in elevated CO_2_ treatment, but the increasing range was relatively flat. No significant warming effects on *E. urophylla* soil microbial biomass could be detected, whereas total PLFAs and PLFAs of various microorganisms of *A. mearnsii* soil were slightly reduced in HA compared to LA (Additional file 1: Figure S4a). With regard to the changes of the biomass of the four categories of microorganisms in the +RE treatment soil were shown in Additional file 1: Figures S3b and S4b. The variation tendency was similar with bare soil treatment, that biomass of G+, G- and F increased significantly under elevated CO_2_ condition (LE and HE). But the magnitude of changes was obviously smaller. In comparison, the biomass of B, F and A in the planted treatment (Additional file 1: Figures S3c and S4c) did not change (*E. urophylla*) significantly and significantly decreased (*A. mearnsii*) under warming and elevated CO_2_ condition, respectively.

**Fig. 3:**
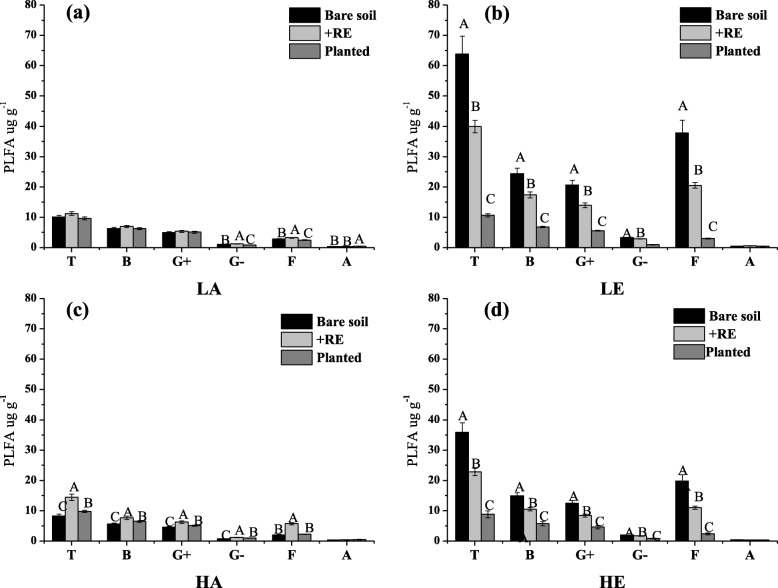
PLFAs of different microbial communities of *Eucalyptus urophylla* soil. **a** Lower temperature& Ambient CO_2_, **b** Lower temperature& Elevated CO_2_, **c** Higher temperature& Ambient CO_2_, and **d** Higher temperature& Elevated CO_2_. For each microbial group (Total (T), Bacteria (B), Gram positive bacteria (G+), Gram negative bacteria (G-), Fungi (F) and Archaea (A), data of mean values and standard deviations of three replicates are presented. Significant differences of variable means among different treatments at each sampling date are indicated by different letters (*P* < 0.05)

**Fig. 4:**
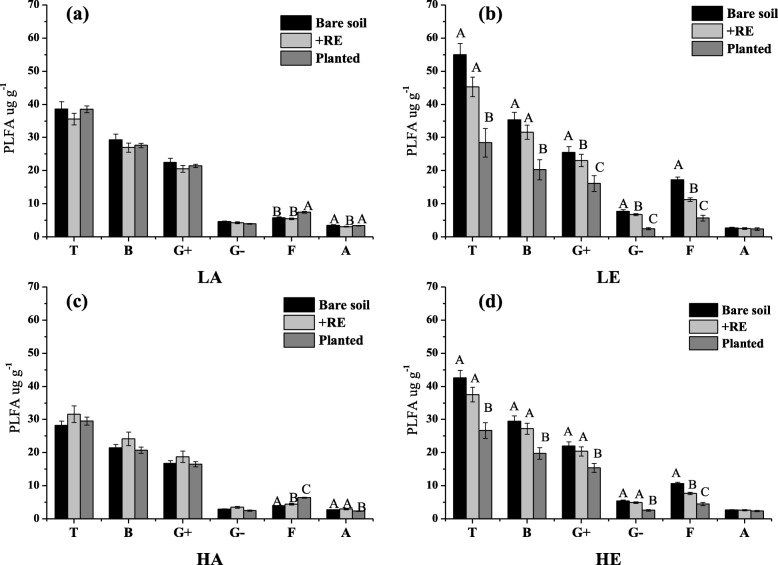
PLFAs of different microbial communities of* Acacia mearnsii* soil. **a** Lower temperature& Ambient CO_2_, **b** Lower temperature& Elevated CO_2_, **c** Higher temperature& Ambient CO_2_, and **d** Higher temperature& Elevated CO_2_. For each microbial group (Total (T), Bacteria (B), Gram positive bacteria (G+), Gram negative bacteria (G-), Fungi (F) and Archaea (A), data of mean values and standard deviations of three replicates are presented. Significant differences of variable means among different treatments at each sampling date are indicated by different letters (*P* < 0.05)

**Fig. 5:**
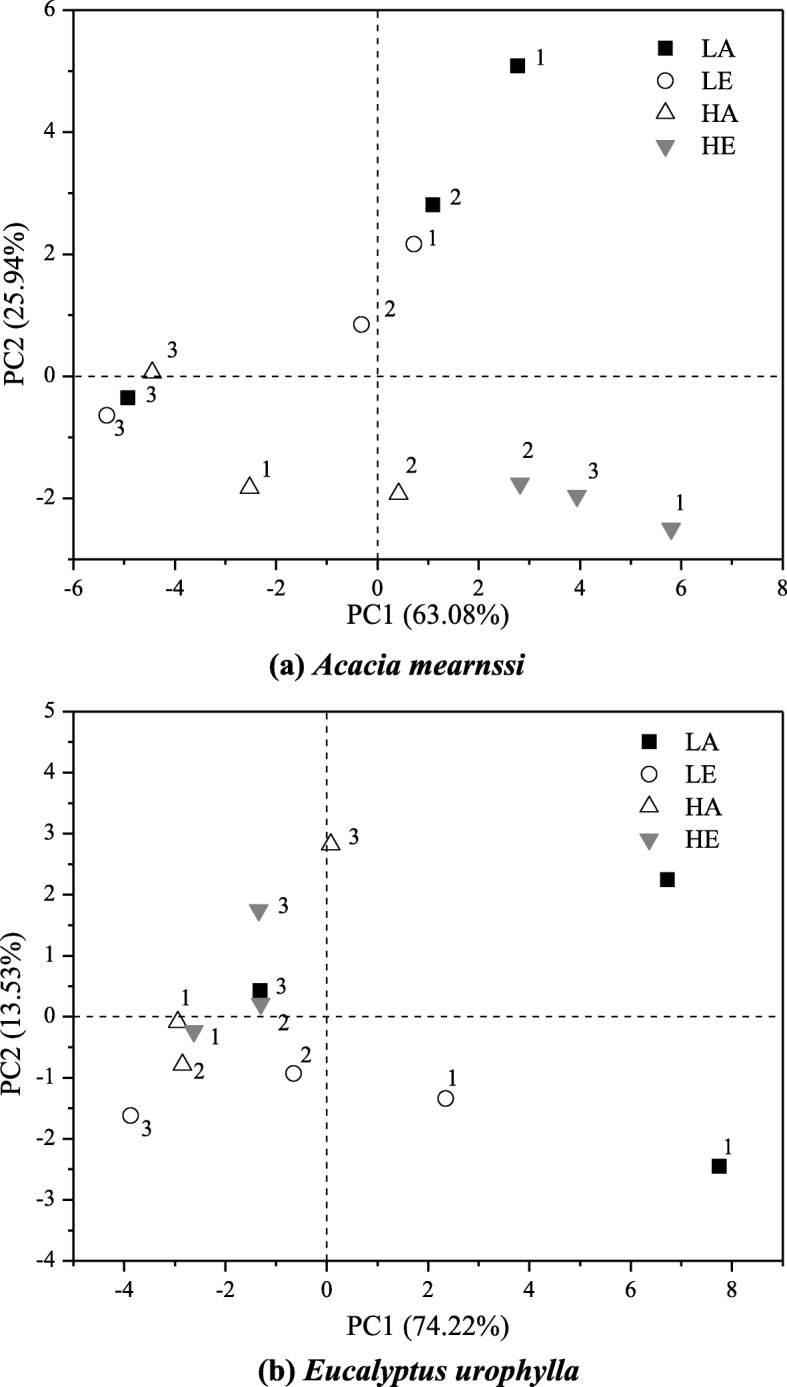
Soil microbial community structure of *Acacia mearnsii* (**a**) and *Eucalyptus urophylla* (**b**)

According to the results of three treatments, we found that the indirect effects of warming and elevated CO_2_ on soil microbial abundance via plant root secretion of two plant spicies studied here were mostly negative. Under LA condition, +RE and planted treatment did not significantly affect biomass of bacteria, fungi and actinomycete in both *A. mearnsii* and *E. urophylla* soil compared to bare soil treatment (Figs. 3a and 4a). However, as shown in Figs. 3b and 4b, +RE and planted significantly reduced biomass of total and various microorganisms compared to bare soil plot under elevated CO_2_ condition, which proved that elevated CO_2_ could affect soil microbial communities via plant root exudates indirectly. Conversely, biomass of various microorganisms (G^+^, G^−^, F) significantly increased in +RE and planted of *E. urophylla* plot at warming chamber (Fig. 3c). No significant *A. mearnsii* root secretion effects on microbial biomass could be found in warming chamber (Fig. 4c). The soil microbial structure determined through PCA of the PLFAs data showed the distribution on the PC axis was significantly different (Fig. 5). Three treatments (1: bare soil; 2: +RE; 3: planted) of LE and HE were dispersed over the PCA plot, also indicating that elevated CO_2_ could change microbial community structure via plant root secretion indirectly.

## Discussion

Root exudates exhibit high variability within different plant species and even cultivars, within different developmental stages and environmental conditions of individual plants [30, 31]. The results presented here demonstrated that the composition and proportions of root exudates secreted by *E. urophylla* and *A. mearnsii* were different, but the main compounds were both the organic acids. As the most widely studied component of root exudates, diverse array of organic acids have been identified and their functions in the rhizosphere have been elucidated [32]. Most of low-molecular-weight organic acids, like glutaric, oxalic, malonic have been found to be nutrient sources of rhizosphere microorganisms [33, 34]. And like aldonic, malic, erythronic have been regarded as the chemoattractant signals to microbes. In this study, dominant organic acid components secreted by *A. mearnsii* were palmitic, stearic, and of *E. urophylla* were palmitic, benzoic, vanillic, ect. These high molecular weight organic acids were reported as common allelopathic chemicals, or phytoalexins against soil pathogens [20, 21, 35].

Many studies demonstrated that adequate climate warming and enriched CO_2_ stimulate secretion of root exudates with their chemical composition changed [14, 31, 36]. The plant photosynthetic capacity increases with warming and elevated CO_2_ concentration, accordingly the content of root exudates as the products of photosynthesis would increase [1, 36] Our results showed that warming and elevated CO_2_ increased significantly the root exudation in both tree species, but the stimulated effects were markedly greater in *A. mearnsii* compared to *E. urophylla*. Although the exact mechanisms behind these variations are still not clear, we propose several possible potential mechanisms to explain this observation. Primarily, the differences in root morphological traits between the two plants could be largely responsible for the variations. *A. mearnsii* seedlings had evident higher root length than that of the *E. urophylla* at the beginning of our experiment. Root length of *A. mearnsii* was further enhanced by experimental warming and elevated CO_2_ through observation. The positive correlation between the root exudates and plant root length had been proved in a previous study [37]. In addition, the higher effects of experimental warming on the root exudates of *A. mearnsii* might be related to environmental adaptation. Compared to *E. urophylla, A. mearnsii* seedlings were living in a lower temperature environment (mean annual temperature is 14.5 °C), so that it was more sensitive to the experimental warming. However, the deep mechanism is still largely unclear and represents a critical area for further study.

In order to simulate anticipated global warming (1–5 °C), a large array of field and lab warming studies have been performed. A common finding of most these warming studies was that warming did not significantly increase microbial biomass in soil [6, 7, 38]. Our results also clearly show that 6°Cwarming (normal CO_2_ concentration) did not affect microbial biomass and community composition of the *E. urophylla* soil, but significant reduced microbial biomass of *A. mearnsii* soil, which showed microorganisms of *A. mearnsii* soil were more sensitive to warmer conditions.

As it has been reported that soil stores 2500 Pg of carbon (C) which is significantly more than the C locked in atmospheric pools [39], the effects of elevated CO_2_ on soil microbial communities were mostly deemed to indirectly, that through increasing plant biomass and belowground carbon inputs, which led to significant changes of the microenvironment [40]. But interestingly, we not only found the negative indirect effects, but also found that elevated CO_2_ could directly change the community structure of microorganism and significantly increased the microbial biomass of soil in both *E. urophylla* and *A. mearnsii*. Similarly, some studies found elevated CO_2_ significantly increased the biomass of soil in non-rhizosphere soil or bulk soil [41, 42]. Although the exact mechanisms behind the direct effect are not well known, we believe that the ratio of gases in the atmosphere may cause the changes in soil surface moisture or soil respiration. Indeed, during experiment, we found that the surface of the soil in elevated CO_2_ chamber get wetter transitorily, although we strictly maintained the water quantity and air humidity of each treatment. It is speculated that elevated CO_2_, associated greater soil moisture, led to significant changes in the structure of soil microbial communities, stimulation of microbial biomass, especially fungi growth. There were lots of studies dealing with the effects of elevated CO_2_ on soil microbial communities in various ecosystems, which obtained different results. The differences could be due to varying response time, different ecosystems, plant species and microbial groups. For instance, while some studies have shown that microbial numbers, metabolic activity, or biomass can increase in rhizosphere or bulk soils exposed to elevated atmospheric CO_2_ [9, 41, 43], others have not shown such a response. After investigating soil microbial community structure of a species-rich semi-natural calcareous grassland that had been exposed to elevated CO_2_ for 6 growing seasons, Ebersberger et al. [44] observed that PLFA profiles were not affected by CO_2_ enrichment and the ratio of fungal and bacterial PLFA did not change. Microbial responses to elevated CO_2_ are specific microbial groups. Lee et al. [45], using a FACE field experiment in in salt marsh ecosystem, found that elevated CO_2_ and N addition increased the abundance in fungi and archaea, but not in bacteria. Moreover, microbial responses were highly correlated with CO_2_ concentration, like Ma et al. [46] found that CO_2_ concentration from normal atmosphere to 10,000 μmol/mol, some new bacterial populations appeared resulted in an increase in soil bacterial diversity, When the CO_2_ concentration was greater than 10,000 μmol/mol, elevated CO_2_ caused the bacteria that did not adapt to high concentration CO_2_ in the sorghum rhizosphere soil to die. Moreover, biodiversity and function would respond differently depending on whether a community was exposed to an abrupt or a gradual increasing CO_2_. Klironomos et al. [47] tested on a mycorrhizal fungal community over a period of 6 years, and found that studies may overestimate some community responses to increasing CO_2_ because biota may be sensitive to ecosystem changes that occur as a result of abrupt increases. In this study, we investigated soil microbial community structure of two plant species that had been exposed to elevated CO_2_ (600 ppm) for 6 months, observing that elevated CO_2_ had no significant effect on microbial biomass and soil microbial community structure of planted treatment, which indicated that there was an inhibitory effect of seedling to soil microorganisms that balanced the direct promotion effect mentioned above. As root exudates are considered to be one of the most important factors that affect soil microorganisms [20], the organic acids existed in root exudates have been demonstrated as putative allelochemicals, which exhibit the potential for toxicity to no matter plant or microorganisms [48]. There were 14 and 11 organic acids compounds identified from root exudates of *E. urophylla* and *A. mearnsii*, dominant including palmitic acid, stearic acid, benzoic acid, etc., which were previously reported as antimicrobial chemicals [21, 35]. Yang et al. [48] and Ibrahim [49] Found that palmitic acid and liposomal lauric acids had inhibitory effects on *Propionibacterium acnes* and some Gram-negative bacteria. Zhou and Wu [50] also found that the addition of p-coumaric acid significantly increased the abundance of rhizospheric bacterial and fungal community when the additive concentrations exceeded 1 mM. Our study showed that soil microbial biomass significantly decreased in soil adding root exudates, which containing high contents of organic acids, supported the antibacterial effect of organic acids to a certain degree.

The impact of organic acids on soil microorganisms shows a concentration effect. Qu and Wang [51] found that the addition of phenol 2,4-di-tert-butylphenol and vanillic acid resulted in stimulation of microbial biomass at low concentrations and inhibition at high concentrations. In this study, elevated CO_2_ increased significantly the root exudation of organic acids, associated with the reduction of biomass of bacteria and fungi in soil, which may support the “low promotion and high suppression” effect of phenolic acids on soil microbes.

In this study, warming and elevated CO_2_ directly affected plant root secretion and soil microbes. Compared to elevated temperature, elevated CO_2_ had stronger effect on increasing soil microbial biomass. However, plant roots could significantly increase the secretion of antibacterial chemicals, which significantly inhibited the biomass of bacteria and fungi in elevated CO_2_ environment. For planted treatment, this inhibitory effect counteracted the promotion from elevated CO_2_ on soil microbial biomass, and resulted in no significant impact on microbial biomass. The results showed that climate change can directly affect the microbial biomass, as well as indirectly affecting the soil microbial community structure – mediated by altered composition and content of plant root exudates. The mechanism responsible for the effect of root exudates on soil microorganisms is likely from the action of the constituent organic acids.

## Conclusion

The phenomena that elevated temperature directly decreased biomass of soil-borne bacteria and fungi for *A. mearnsii*, and elevated CO_2_ increased soil microbial biomass for both *A. mearnsii* and *E. urophylla*, demonstrated that climate change can directly affect microorganisms. In elevated CO_2_ environment, plant roots could significantly increase the secretion of antibacterial chemicals inhibiting the growth of bacteria and fungi. This indicated that climate change can indirectly affect the soil microbial community structure by changing the composition and content of plant root exudates.

## Methods

### Study site

Soil sample in *Eucalyptus urophylla* S.T. Blake plantation was taken from Shuilianshan Forest Park (22°58′N, 113°42′E) of Dongguan city, Guangdong Province, China, with a permission from Administration of Dongguang Botanical Garden. This region has a subtropical monsoon maritime climate. The average annual temperature is 23.2 °C, mean annual precipitation is 1780.4 mm and the rainy season is during April–September. The soils of the study area were classified as ferralsol. Vegetation in the zone is subtropical evergreen broad-leaved forest, but trees in the *E. urophylla* plantation were over 17 years old and their average height exceeded 12 m, with a few shrubs and herbaceous plants in their understory [23]. Seedlings of *E. urophylla* (40–50) cm in height were obtained from Xinlvmiaomu Company, Guangzhou.

Seedlings of *Acacia mearnsii* De Willd (of 30–40 cm in height) and an accompanying soil sample were taken from Mingfengshan Forest Park of Kunming city, Yunnan Province, China, with a permission from Administration of Mingfengshan Forest Park. The climate of the region is subtropical low-latitude plateau monsoon, mean annual temperature is 14.5 °C and the maximum height above sea level is 2068 m. The soils of study area were classified as acrisols. Vegetation in the area was dominated by Fagaceae, Lauraceae and Magnoliaceae.

Voucher specimen of *E. urophylla* and *A. mearnsii* were not deposited in this study since they are the most common trees in Southern China.

### Experiment in growth chambers

We sampled and characterized root exudates from hydroponic cultivation solution [52, 53]. Besides, considering simulating the real state of effects of climate warming on the plant-soil system, we set up three treatments: (1) bare soil (direct effects of warming and elevated CO_2_), (2) soil added with root exudates solution form hydroponic cultivation (demonstrating the indirect effect of root exudates) and (3) soil planted with seedling (integral direct). The detailed experimental steps are described below.

The experiment was conducted during a 26-week period from June 2014 to December 2014. Plant pots, 20 cm in diameter, each pot was filled with 8 kg soil collected from the *E. urophylla* and *A. mearnsii* plantations respectively, and divided into three treatments as mentioned above: bare soil, soil with added root exudates (+RE) and soil planted with seedling (Planted).

All treatments were triplicated. Seedlings of *E. urophylla* and *A. mearnsii* were surface sterilized with 1% sodium hypochlorite solution for 5 min and rinsed three times in sterile distilled water. One seedling was transplanted into planted treatment pot and another to a 250-ml glass bottle which filled with 200 ml Hoagland nutrient solution [54], to collect root exudates solution for the +RE treatment. The root exudates solution was sampled once for every 3 days throughout the experiment period, and then divided into two parts, one half was added to +RE treatment pots, and another was stored at − 20 °C for chemical compounds analysis. New sterile nutrient solutions were added after each sampling. Other two treatments (bare soil, planted) were added with same volume of hoagland nutrient solution simultaneously to keep the same soil moisture.

As shown in Table 1, the three treatments were subjected to four different combinations of temperature and CO_2_ concentration, each combination of treatments was applied in a separate isolated artificial climatic chamber (one per combination). Two day/night temperature regimes was set: lower temperatures (26/19 °C) and elevated temperatures (32/25 °C), which according to simulate the distant future climate projections [1]. To assess the effect of changes in the CO_2_ concentration, each temperature treatment was combined with two CO_2_ concentration regimes of ambient CO_2_ (380 ppm) and elevated CO_2_ (600 ppm). All pots were randomly placed in the artificial climatic chambers. During the cultivation, 60% of water-holding capacity of the soil was maintained and water loss was compensated using sterile distilled water.

**Table 1:** The design of environmental impact factors

Design factors	Elevated CO_2_(600 ppm)			Ambient CO_2_(380 ppm)		
	Bare soil	+RE	planted	Bare soil	+RE	planted
Higher temperature(32/25 °C)	HE1	HE2	HE3	HA1	HA2	HA3
Lower temperature(26/19 °C)	LE1	LE2	LE3	LA1	LA2	LA3

After 26 weeks of growth, the 5-10 cm top soil in the middle of each pot was carefully collected (for planted treatment pot, 5–10 cm rhizosphere soil was collected), mixed, homogenized to obtain 0.5 kg soil pot^− 1^, and stored at 4 °C for soil microbial community structure analysis.

### Chemical compounds of root exudates

An XAD-4 resin column (Sigma–Aldrich, St. Louis, MO, USA) was used to capture the target compounds of root exudates from stored nutrient solution. The column was washed with ten-bed volume eluates of hexane, ethyl acetate, methanol and distilled water (1:1:2:1). The elution solution was filtered and concentrated to 1 mL in a rotary evaporator under reduced pressure at 40 °C [7]. Finally, after the derivatization pretreatment (derivatized with 200 μL methyl cyanide and 200 μL BSTFA+ 1% TMCS), the samples were analyzed by a gas chromatography (Agilent 7890, California, USA) coupled with a mass selective detector (Agilent 5975), helium as the carrier gas at a flow rate of 1 mL/min. The samples (1 μL) were injected in the split mode (1:50) and isolated on an HP-5MS column (30 m × 0.25 mm × 0.25 μm, Agilent) with the following temperature program: initial temperature of 50 °C, rising at a rate of 6 °C/min to 150 °C with a 1-min hold, and then increased to 220 °C, at rate of 8 °C/min with a hold for 1minfollowed by a rise to 280 °C at a rate of 20 °C/min.

### Composition of soil microbial community structure

We analyzed the structure of microbial communities in soil samples using phospholipid fatty acid (PLFA) analysis with a method adapted from previous study [55]. In brief, 10 g of soil sample was extracted with a one-phase solvent mixture of chloroform, methanol and 50 mmol/L phosphate buffer (pH 7.4) (1:2:0.8 by volume) for 24 h. After 1 h centrifugation, the supernatant was removed. Then the remaining soil was re-extracted for 12 h. After re-centrifugation, the supernatant was removed and the combined extract was then evaporated by N_2_ to a volume of 1 mL. The polar lipids were then subjected to saponification and methylation for gas chromatograph–mass spectrometer (GC-MS). PLFA peaks were identified by comparing their retention times and mass spectra with those from reference compounds (a 37-component fatty acid methyl ester mix (Nu-chekprep, USA), and a C19 methyl ester standard (Nu-chekprep, USA) from the NIST 2011 spectral database. For characterizing the community structure we used the terminal-branched saturated PLFA peaks i14:0, i15:0, a15:0, i16:0, a16:0, i17:0, a17:0, c17:0 as marker for Gram-positive bacteria. The mono-unsaturated and cyclopropyl saturated peaks 16:1ω5, 16:1ω7, 17:1ω7, 18:1ω11, cy19:0 were used as indicators for Gram-negative bacteria and the PLFA peaks 14:0, 15:0, 17:0 for unspecific bacteria. Furthermore, 18:1ω9c, 18:2ω6,9, 18:3ω6,9,12 was used as fungal PLFA marker. The methylic, mid-chain-branched saturated PLFA peaks 10Me16:0,10Me17:0, 10Me18:0 were used as indicators for actinomycetes.

### Data analysis

The differences of root exudates between two species under four environmental conditions were analyzed with the principal component analysis (PCA). Differences of soil microbe groups among different treatments were compared with one-way ANOVA and the least significant difference at 95% confidence level (*p* < 0.05). Soil microbial community structures were also compared with PCA. The analyses were performed using SPSS 16.0 software.

## Supplementary information

**Additional file 1: Table S1**. Compounds of four kinds of root exudates from *A. mearnsii*. **Table S2.** Compounds of four kinds of root exudates from E.urophylla. **Figure S1.** Principal component analysis of 39 compounds for *Acacia mearnsii*. **Figure S2.** Principal component analysis of 35 compounds for E.urophylla. **Figure S3.** PLFAs of different microbial communities of Eucalyptus urophylla soil. **Figure S4.** PLFAs of different microbial communities of *Acacia mearnsii*. (DOCX 160 kb)

## Data Availability

The datasets generated during and/or analysed during the current study are available from the corresponding author on reasonable request.

## Abbreviations

ANOVA: analysis of variance
BSTFA: Bistrifluoroacetamide
CO_2_: carbon dioxide
GC-MS: gas chromatograph–mass spectrometer
IPCC: Intergovernmental Panel on Climate Change
PC: principal component
PCA: principal component analysis
PGPR: plant growth promoting rhizobacteria
PLFA: phospholipid fatty acid
PTFE: polytetrafluoroethylene
SOM: soil organic matter
TMCS: templated mesoporous carbons
