# Supplementary material for: Effect of root exudates of Eucalyptus urophylla and Acacia mearnsii on soil microbes under simulated warming climate conditions
Source: BMC Microbiol. 2019 Oct 15;19:224. doi: 10.1186/s12866-019-1604-6 (PMC6794899; doi:10.1186/s12866-019-1604-6)
Supplement: Supplementary file 1 — Additional file 1: Table S1. Compounds of four kinds of root exudates from A. mearnsii. Table S2. Compounds of four kinds of root exudates from E.urophylla. Figure S1. Principal component analysis of 39 compounds for Acacia mearnsii. Figure S2. Principal component analysis of 35 compounds for E.urophylla. Figure S3. PLFAs of different microbial communities of Eucalyptus urophylla soil. Figure S4. PLFAs of different microbial communities of Acacia mearnsii. (DOCX 160 kb) [file 12866_2019_1604_MOESM1_ESM.docx]

**SUPPLEMENTARY MATERIALS**

Table S1 Compounds of four kinds of root exudates from *A. mearnsii*

| Class | English Name | Molecular Formula | Peak Area | | | |
| --- | --- | --- | --- | --- | --- | --- |
|  |  |  | LE | HE | HA | LA |
| phenol | 2,6-Xylenol, 4-nitro | C_8_H_9_NO_3_ | / | 4.12×10^6^±3.50×10^5^ | 2.42×10^6^±2.31×10^5^ | 2.06×10^6^±1.96×10^5^ |
|  | 2,4,6-trimethylpyridine | C_6_H_6_ClNO | 1.13×10^7^±9.99×10^5^ | 1.47×10^7^±1.31×10^6^ | 8.69×10^6^±7.42×10^5^ | 8.92×10^6^±7.62×10^5^ |
|  | 2,4-Ditertbutyl phenol | C_14_H_22_O | 5.78×10^6^±6.67×10^5^ | 4.94×10^6^±6.68×10^5^ | 5.59×10^6^±3.92×10^5^ | 5.94×10^6^±4.16×10^5^ |
| Alkane | Butane | C_4_H_10_ | 2.76×10^7^±2.13×10^6^ | 2.67×10^7^±2.59×10^6^ | 1.62×10^7^±6.19×10^5^ | 1.69×10^7^±6.68×10^5^ |
|  | 2-Methyldecane | C_11_H_24_ | 7.55×10^6^±7.90×10^5^ | 8.23×10^6^±8.34×10^5^ | 3.81×10^6^±4.15×10^5^ | 3.96×10^6^±4.29×10^5^ |
|  | Dodecane | C_12_H_26_ | 3.65×10^6^±3.30×10^5^ | / | / | / |
|  | N-Tridecane | C_13_H_28_ | 2.86×10^6^±2.71×10^5^ | 3.75×10^6^±4.09×10^5^ | / | 1.83×106±2.30×10^5^ |
|  | N-tetradecane | C_14_H_30_ | 3.43×10^6^±3.79×10^5^ | 3.21×10^6^±3.52×10^5^ | 3.82×10^6^±3.69×10^5^ | 3.90×10^6^±4.07×10^5^ |
|  | Heptadecane | C_17_H_36_ | 2.17×10^6^±2.61×10^5^ | 2.68×10^6^±3.08×10^5^ | / | / |
|  | Octadecane | C_18_H_38_ | 1.52×10^6^±2.02×10^5^ | 2.24×10^6^±2.68×10^5^ | / | / |
|  | N-Heneicosane | C_21_H_44_ | 1.84×10^6^±2.23×10^5^ | / | / | / |
|  | Pentacosane | C_14_H_22_O | 1.87×10^6^±2.26×10^5^ | 2.71×10^6^±3.12×10^5^ | 1.65×10^6^±2.25×10^5^ | 1.97×10^6^±2.43×10^5^ |
|  | Octacosane | C_28_H_58_ | / | 2.26×10^6^±2.70×10^5^ | / | / |
| Organic acids | DL-Pipecolinic acid | C_6_H_11_NO_2_ | 4.96×10^6^±5.17×10^5^ | 5.47×10^6^±5.71×10^5^ | 3.02×10^6^±2.83×10^5^ | 3.18×10^6^±3.55×10^5^ |
|  | Propanedioic acid | C_3_H_4_O_4_ | / | 1.52×10^6^±2.02×10^5^ | / | / |
|  | Benzoic acid | C_7_H_6_O₂ | 4.59×10^6^±6.23×10^5^ | 1.89×10^6^±2.43×10^5^ | / | 2.43×10^6^±2.93×10^5^ |
|  | Decanoic acid | C_10_H_20_O_2_ | 1.12×10^6^±1.97×10^5^ | / | 1.07×10^6^±1.88×10^5^ | 1.15×10^6^±2.06×10^5^ |
|  | 2-Thiophenecarboxylic acid | C_5_H_4_O_2_S | 4.25×10^6^±5.81×10^5^ | 4.89×10^6^±5.71×10^5^ | 2.88×10^6^±3.93×10^5^ | 3.03×10^6^±4.18×10^5^ |
|  | Cinnamic acid | C_9_H_8_O_2_ | 2.78×10^6^±3.98×10^5^ | 3.08×10^6^±4.15×10^5^ | 1.18×10^6^±2.00×10^5^ | 1.29×10^6^±2.21×10^5^ |
|  | 6-Chloro-N-Hexanoic Acid | C_6_H_11_ClO_2_ | / | 6.46×10^6^±8.03×10^5^ | 7.77×10^6^±1.90×10^4^ | / |
|  | Myristic acid | C_14_H_28_O_2_ | 1.94×10^7^±1.08×10^6^ | 2.34×10^7^±2.59×10^6^ | 3.41×10^7^±4.30×10^6^ | 1.29×106±2.12×10^5^ |
|  | Palmitic acid | C_16_H_32_O_2_ | 3.28×10^7^±1.24×10^6^ | 3.55×10^7^±4.16×10^6^ | 2.60×10^7^±3.06×10^6^ | 2.43×10^7^±2.86×10^6^ |
|  | Octadecanoic acid | C_18_H_36_O_2_ | 1.49×10^7^±6.04×10^5^ | 2.59×10^7^±2.99×10^6^ | 2.03×10^7^±2.98×10^6^ | 1.81×10^7^±2.14×10^6^ |
|  | Docosanoic acid | C_22_H_44_O_2_ | 8.51×10^6^±1.38×10^6^ | 1.07×10^7^±1.78×10^6^ | / | / |
| ester | Dioctyl phthalate | C_24_H_38_O_4_ | 4.27×10^6^±5.93×10^5^ | 5.13×10^6^±5.40×10^5^ | 8.80×10^6^±1.24×10^6^ | 1.09×10^7^±1.08×10^6^ |
|  | Palmitic acid ethyl ester | C_18_H_36_O_2_ | 9.03×10^6^±9.10×10^5^ | 1.33×10^7^±1.32×10^6^ | / | / |
|  | Isopropyl stearate | C_21_H_42_O_2_ | / | / | 2.31×10^6^±3.63×10^5^ | 2.26×10^6^±2.69×10^5^ |
|  | Lupeol acetate | C_32_H_52_O_2_ | 9.35×10^6^±9.40×10^5^ | 1.61×10^7^±1.59×10^6^ | 1.72×10^7^±2.37×10^6^ | / |
| alkene | Alpha-phellandrene | C_10_H_16_ | 1.99×10^7^±2.30×10^6^ | 1.24×10^7^±1.44×10^6^ | 3.72×10^6^±6.84×10^5^ | 5.63×10^6^±5.87×10^5^ |
|  | (+)-Aromadendrene | C_15_H_24_ | 1.84×10^7^±1.80×10^6^ | 9.96×10^6^±8.02×10^5^ | / | 5.53×10^6^±5.78×10^5^ |
|  | Artemisia triene | C_10_H_16_ | 6.82×10^6^±9.56×10^5^ | 9.65×10^6^±1.24×10^6^ | 3.91×10^6^±5.77×10^5^ | 3.61×10^6^±3.96×10^5^ |
|  | 3-Methylene-1,5,5-trimethylcyclohexene | C_10_H_16_ | 2.91×10^6^±2.75×10^5^ | 4.92×10^6^±4.24×10^5^ | 1.64×10^6^±2.74×10^5^ | 1.75×10^6^±2.22×10^5^ |
| alcohol | Benzylalcohol | C_7_H_8_O | 1.17×10^6^±1.25×10^5^ | / | 3.27×10^6^±4.91×10^5^ | 3.59×10^6^±3.67×10^5^ |
|  | Glycerin | C_3_H_8_O_3_ | 4.46×10^6^±3.89×10^5^ | 1.48×10^7^±1.46×10^6^ | 2.95×10^6^±4.49×10^5^ | 3.08×10^6^±3.46×10^5^ |
|  | (±)-3,7-dimethyl-octa-1,6-dien-3-ol | C_10_H_18_O | 3.70×10^6^±4.04×10^5^ | 5.55×10^6^±5.79×10^5^ | / | / |
|  | meso-Erythritol | C_4_H_10_O_4_ | 2.70×10^6^±3.10×10^5^ | 2.50×10^6^±3.90×10^5^ | 4.93×10^6^±7.07×10^5^ | 6.00×10^6^±8.47×10^5^ |
|  | Ribitol | C_5_H_12_O_5_ | 4.08×10^6^±4.40×10^5^ | 2.79×10^6^±3.19×10^5^ | / | 3.66×10^6^±4.01×10^5^ |
| others | N-trifluoroacetylmorpholine | C_6_H_8_F_3_NO_2_ | / | / | 2.39×10^6^±2.81×10^5^ | 2.58×10^6^±3.00×10^5^ |
|  | 1,5-Diphenyl-1,5-Pentanedione | C_17_H_16_O_2_ | / | / | 2.53×10^6^±2.95×10^5^ | / |

Table S2 Compounds of four kinds of root exudates from *E．urophylla*

| Class | English Name | Molecular Formula | Peak Area | | | |
| --- | --- | --- | --- | --- | --- | --- |
|  |  |  | LE | HE | HA | LA |
| phenol | 3-Acetoxypyridine | C_7_H_7_NO_2_ | / | 8.88×10^6^±2.34×10^6^ | 6.33×10^6^±5.87×10^5^ | / |
|  | 1-Hydroxy-2-tert-butyl-4-methylbenzene | C_11_H_16_O | 5.86×10^6^±1.22×10^6^ | 4.24×10^6^±3.93×10^5^ | 3.51×10^6^±9.26×10^5^ | 3.02×10^6^±1.^6^×10^6^ |
|  | 2,6-Di-tert-butylphenol | C_14_H_22_O | 1.25×10^7^±1.15×10^6^ | 1.43×10^7^±2.29×10^6^ | 9.68×10^6^±1.00×10^6^ | 1.08×10^7^±9.98×10^5^ |
| Alkane | Heptadecane | C_17_H_36_ | 1.59×10^7^±1.47×10^6^ | 1.31×10^7^±1.20×10^6^ | 2.10×10^6^±1.95×10^5^ | 5.21×10^6^±3.83×10^5^ |
|  | Octadecane | C_18_H_38_ | 7.74×10^6^±7.17×10^5^ | 8.57×10^6^±8.48×10^5^ | / | 3.40×10^6^±3.15×10^5^ |
|  | Eicosane | C_20_H_42_ | 2.36×10^6^±3.55×10^5^ | 2.30×10^6^±2.39×10^5^ | / | / |
|  | Heneicosane | C_21_H_44_ | 1.13×10^7^±1.69×10^6^ | 6.78×10^6^±6.28×10^5^ | 3.55×10^6^±4.92×10^5^ | 4.63×10^6^±4.29×10^5^ |
|  |  |  | 8.23×10^6^±8.14×10^5^ | 9.11×10^6^±8.44×10^5^ | / | 4.83×10^6^±4.48×10^5^ |
| Organic acids | Benzoic acid | C_7_H_6_O₂ | 3.24×10^7^±4.87×10^6^ | 2.67×10^7^±5.55×10^6^ | 3.13×10^6^±4.71×10^5^ | 1.50×10^7^±4.45×10^6^ |
|  | Phthalic acid | C_8_H_6_O_4_ | 5.55×10^6^±5.14×10^5^ | 6.33×10^6^±5.87×10^5^ | 3.86×10^6^±3.57×10^5^ | / |
|  | P-hydroxybenzoic | C_7_H_6_O_3_ | 2.16×10^7^±3.74×10^6^ | 1.34×10^7^±2.16×10^6^ | 1.^6^×10^7^±1.10×10^6^ | 8.93×10^6^±8.28×10^5^ |
|  | vanillic acid | C_8_H_8_O_4_ | 1.84×10^7^±1.71×10^6^ | 1.17×10^7^±1.75×10^6^ | 6.57×10^6^±1.73×10^6^ | 7.^6^×10^6^±8.68×10^5^ |
|  | cinnamic acid | C_9_H_8_O_2_ | 2.17×10^6^±2.01×10^5^ | 1.83×10^6^±6.88×10^5^ | 1.39×10^6^±1.29×10^5^ | 1.33×10^6^±4.67×10^5^ |
|  | Fumaric acid | C_4_H_4_O_4_ | 4.93×10^6^±2.24×10^5^ | 2.89×10^6^±2.27×10^5^ | / | / |
|  | 2-Hexadecenoic acid | C_16_H_30_O_2_ | 6.11×10^6^±1.86×10^5^ | 3.36×10^6^±3.48×10^5^ | / | 6.69×10^6^±6.20×10^5^ |
|  | Heptadecenoic acid | C_17_H_32_O_2_ | 7.90×10^6^±1.64×10^6^ | 8.79×10^6^±1.32×10^6^ | / | 3.93×10^6^±3.64×10^5^ |
|  | Hexadecanoicacid,2-methyl- | C_17_H_34_O_2_ | 6.78×10^6^±6.28×10^5^ | 6.^6^×10^6^±9.12×10^5^ | / | / |
|  | 9-Octadecenoic acid | C_18_H_34_O_2_ | 1.30×10^7^±7.85×10^5^ | 1.19×10^7^±3.52×10^6^ | 1.33×10^7^±1.64×10^6^ | 1.25×10^7^±1.16×10^6^ |
|  | linoleic acid | C_18_H_32_O_2_ | 3.21×10^6^±3.94×10^5^ | 3.66×10^6^±9.65×10^5^ | 2.37×10^6^±8.92×10^5^ | 2.90×10^6^±1.02×10^6^ |
|  | Lauric acid | C_12_H_24_O_2_ | 5.37×10^6^±9.16×10^4^ | / | 1.18×10^6^±2.45×10^5^ | 1.83×10^6^±3.59×10^5^ |
|  | Palmitic acid | C_16_H_32_O_2_ | 3.66×10^7^±4.50×10^6^ | 3.^6^×10^7^±3.76×10^6^ | 2.03×10^7^±1.88×10^6^ | 2.20×10^7^±6.87×10^6^ |
| ester | Methyl geranate | C_11_H_18_O_2_ | 1.38×10^7^±1.12×10^6^ | 9.28×10^6^±8.60×10^5^ | 6.59×10^6^±1.14×10^5^ | 6.87×10^6^±4.46×10^5^ |
|  | Di-n-butylphthalate | C_19_H_20_O_4_ | 1.93×10^7^±2.91×10^6^ | 9.80×10^6^±8.98×10^5^ | 5.18×10^6^±8.91×10^5^ | 5.71×10^6^±5.29×10^5^ |
|  | 4-Ethylbenzoic acid | C_9_H_10_O_2_ | 5.39×10^6^±1.12×10^6^ | 6.29×10^6^±5.83×10^5^ | / | / |
|  | Dibutyl phthalate | C_16_H_22_O_4_ | 1.16×10^7^±1.^7^×10^6^ | 7.11×10^6^±6.59×10^5^ | 2.82×10^6^±2.61×10^5^ | 4.79×10^6^±1.26×10^6^ |
|  | methyl hexadecanoate | C_17_H_34_O_2_ | 2.41×10^7^±2.24×10^6^ | 1.88×10^7^±2.51×10^6^ | 1.35×10^7^±4.75×10^6^ | 1.46×10^7^±1.35×10^6^ |
|  | Dimethyl Octadecanedioate | C_20_H_38_O_4_ | 2.59×10^6^±2.40×10^5^ | / | 1.59×10^6^±1.47×10^5^ | 2.29×10^6^±2.12×10^5^ |
| alcohol | Benzylalcohol | C_7_H_8_O | 6.58×10^6^±6.10×10^5^ | 7.21×10^6^±6.69×10^5^ | 3.80×10^6^±1.31×10^6^ | 4.31×10^6^±4.00×10^5^ |
|  | Glycerin | C_3_H_8_O_3_ | 6.84×10^6^±2.35×10^6^ | 7.48×10^6^±2.63×10^6^ | 3.86×10^6^±6.68×10^5^ | 3.77×10^6^±3.49×10^5^ |
|  | Ribitol | C_5_H_12_O_5_ | 2.58×10^6^±2.37×10^5^ | 3.78×10^6^±1.42×10^6^ | 1.72×10^6^±1.59×10^5^ | 2.74×10^6^±8.13×10^5^ |
|  | Cyclohexanehexol | C_6_H_12_O_6_ | 7.90×10^6^±7.32×10^5^ | 8.69×10^6^±8.^6^×10^5^ | / | 3.64×10^6^±9.59×10^5^ |
| amine | Oleic acid amide | C_18_H_35_NO | 3.91×10^6^±3.63×10^5^ | 5.54×10^6^±5.^7^×10^5^ | 1.^6^×10^7^±9.86×10^5^ | 5.37×10^6^±4.98×10^5^ |
|  | 9-Octadecenamide | C_18_H_35_ NO | 5.70×10^6^±2.61×10^6^ | 4.96×10^6^±2.27×10^6^ | 8.71×10^6^±2.25×10^6^ | 5.57×10^6^±1.47×10^6^ |
|  | N,N-dimethylhexadecylamine | C_18_H_39_N | 6.98×10^6^±8.58×10^5^ | 6.38×10^6^±1.33×10^6^ | / | 2.98×10^6^±2.76×10^5^ |
| others | 4-Methoxy-3-buten-2-one | C_5_H_8_O_2_ | 6.26×10^6^±5.80×10^5^ | 7.28×10^6^±7.57×10^5^ | 5.43×10^6^±1.87×10^6^ | 6.41×10^6^±5.94×10^5^ |


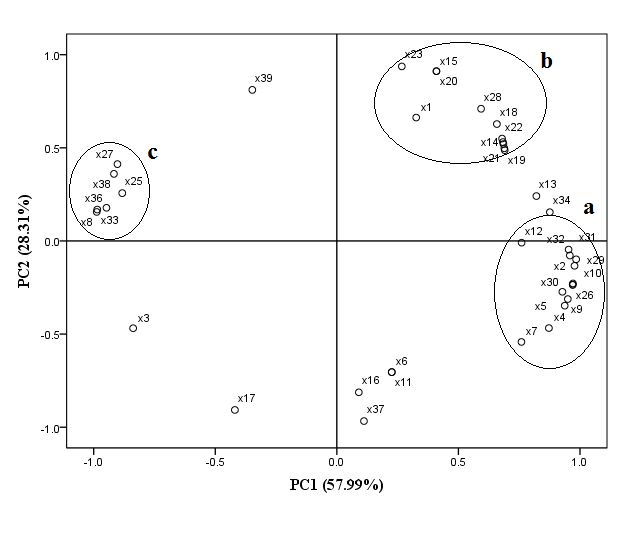


Fig. S1 Principal component analysis of 39 compounds for *Acacia mearnsii*


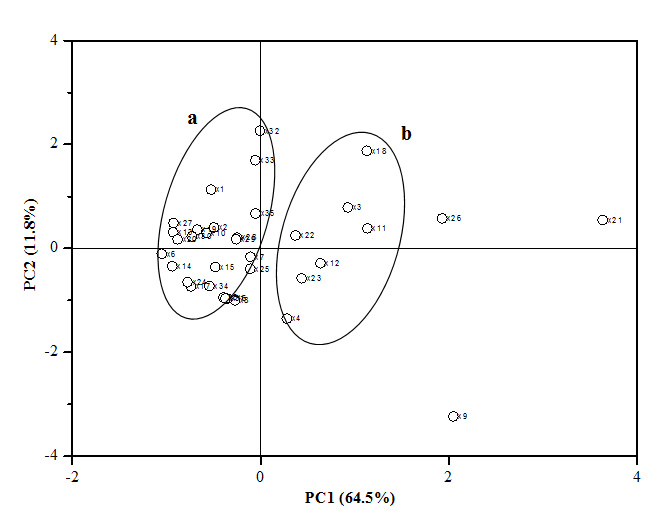


Fig. S2 Principal component analysis of 35 compounds for *E．urophylla*

Fig. S3 PLFAs of different microbial communities of *Eucalyptus urophylla* soil.

For each microbial group (Total (T), Bacteria (B), Gram positive bacteria (G+), Gram negative bacteria (G-), Fungi (F) and Archaea (A), data of mean values and standard deviations of three replicates are presented. Significant differences of variable means among different treatments at each sampling date are indicated by different letters (P < 0.05).

Fig. S4 PLFAs of different microbial communities of *Acacia mearnsii.* Symbols refer to Fig. S3.
